# Low voltage operation of a silver/silver chloride battery with high desalination capacity in seawater†

**DOI:** 10.1039/c9ra02570g

**Published:** 2019-05-14

**Authors:** Pattarachai Srimuk, Samantha Husmann, Volker Presser

**Affiliations:** Department of Materials Science and Engineering, Saarland University 66123 Saarbrücken Germany v.presser@mx.uni-saarland.de; INM - Leibniz Institute for New Materials 66123 Saarbrücken Germany

## Abstract

Technologies for the effective and energy efficient removal of salt from saline media for advanced water remediation are in high demand. Capacitive deionization using carbon electrodes is limited to highly diluted salt water. Our work demonstrates the high desalination performance of the silver/silver chloride conversion reaction by a chloride ion rocking-chair desalination mechanism. Silver nanoparticles are used as positive electrodes while their chlorination into AgCl particles produces the negative electrode in such a combination that enables a very low cell voltage of only Δ200 mV. We used a chloride-ion desalination cell with two flow channels separated by a polymeric cation exchange membrane. The optimized electrode paring between Ag and AgCl achieves a low energy consumption of 2.5 kT per ion when performing treatment with highly saline feed (600 mM NaCl). The cell affords a stable desalination capacity of 115 mg g^−1^ at a charge efficiency of 98%. This performance aligns with a charge capacity of 110 mA h g^−1^.

## Abstract 2

The silver/silver chloride conversion reaction allows for a high desalination capacity of saline media with high molar strength.

## Graphical abstract

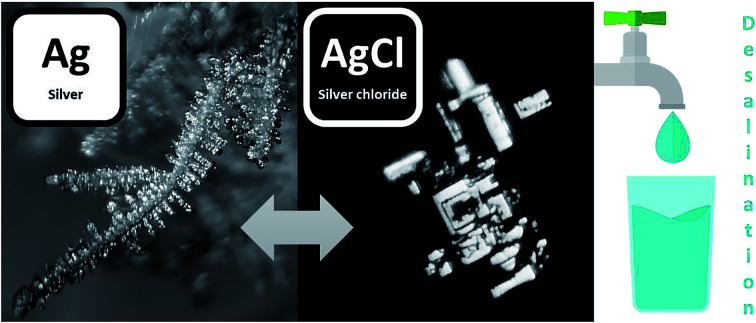

## 1. Introduction

Capacitive deionization (CDI) uses ion electrosorption as a way to immobilize ions at the fluid/solid interface to achieve desalination of a saline medium passing an electrochemical cell.^1–4^ By utilizing the formation of an electrical double-layer, this technology may employ additional ion exchange membranes,^5^ carbon suspension electrodes,^6–8^ or multiple electrolyte flow channels.^9^ CDI can achieve a high charge efficiency, that is, most of the invested charge is used for the ion immobilization (desalination) and not lost to redox-reactions that do not contribute towards the actual desalination process.^10,11^ Using nanoporous carbon and aqueous saline media, one can typically achieve desalination capacities of 10–25 mg g^−1^ (mg NaCl per g of the electrode)^12–16^ at cell voltages of about 1 V for low molar concentrations. State-of-the-art nanoporous carbon materials for CDI electrodes include biomass-derived carbon (ref. 17 and 18) and carbons doped by nitrogen, phosphorus, or sulfur (ref. 19–21).

The mechanism of capacitive deionization requires the electrosorption to be accomplished by preferred counter-ion adsorption, with the immobilization of ions with the opposite charge compared to the electrode. Carbon nanopores lack the required permselectivity; therefore, they are already populated by anions and cations in equal number at the initial uncharged state.^22–24^ Thus, charge storage is initially accomplished by concurrent electro-adsorption of counter-ions and ejection of co-ions, which have the same charge sign as the carbon pore wall. Consequently, desalination is only accomplished after the population of co-ions has been depleted; this can be achieved at low and ultralow molar strength, like for brackish water with salinity below 50 mM.^25,26^ This limitation still applies to capacitive deionization with nanoporous carbon, but it is increased when using materials capable of other charge storage mechanisms, such as ion insertion (*e.g.*, sodium manganese oxide,^27^ hexacyanoferrates,^28^ or 2D layered materials^25,29^) or reconstitution/conversion reactions (*e.g.*, Ag/AgCl,^27,30^ Bi/BiOCl^31^). Such systems can desalinate highly concentrated saline media (100–900 mM).^32^

The first use of the Ag/AgCl conversion reaction in the context of capacitive deionization, corresponds to the seminal work of Blair and Murphy in 1960, which is considered as the first report of capacitive deionization.^1^ In this work, Blair and Murphy investigated half-cells of carbon and modified carbons, most notably, some of them paired with a silver mesh electrode that was anodized at 2 V in concentrated NaCl aqueous solution. The authors created a hybrid capacitive deionization cell by using a pair of one electrosorptive (capacitive) carbon electrode with one redox-active (faradaic) Ag/AgCl electrode.

After the 1960s, Ag/AgCl resurfaced in the context of electrochemical desalination in the seminal work of Pasta *et al.* in 2012.^27^ In their work, Pasta *et al.* introduced the so-called desalination battery, which is a cell comprised of one electrode capable of selective sodium uptake and another electrode capable of selective chloride uptake. Specifically, the authors paired a Na_2−*x*_Mn_5_O_10_ (NMO) electrode with an Ag electrode to demonstrate the ability of such a cell to effectively desalinate saline media even at molarity typically found for seawater. The combination of Ag/AgCl and NMO was further explored by Chen *et al.* who reported for 15 mM aqueous NaCl (890 ppm) a desalination performance of 57 mg g^−1^ after 100 cycles when sweeping the cell voltage from −1.0 V to +1.5 V.^33^

A novel desalination cell concept, the so-called Na-ion desalination (NID), was introduced by Kyle C. Smith in 2016, with later follow-up work by the same group.^34,35^ In its most simplistic form, the NID concept employs two electrodes of the very same material but with different sodiation states. This can be accomplished, for example, by the use of NMO. During charging, one NMO electrode will uptake sodium ions *via* cation insertion while the other NMO electrode will release sodium from the crystal structure. Feedwater passes by these two electrodes in two flow channels which are separated by an anion exchange membrane. As one channel becomes enriched by sodium deinserted from the NMO, chlorine ions cross the ion exchange membrane into that channel; thereby, the other channel becomes depleted by chlorine and, at the same time, depleted from sodium that is being inserted into NMO. This yields concurrently desalination in one channel and salination in the other flow channel. The NID cell concept can be used also for other materials and material pairings, as also shown by Lee *et al.* where sodium nickel hexacyanoferrate was paired with sodium iron hexacyanoferrate.^36^ The latter configuration was labeled rocking-chair desalination battery and yielded a desalination capacity of about 60 mg g^−1^ in 500 mM NaCl aqueous solution with a cell voltage of 0.8 V. This cell voltage is much lower than what carbon-based CDI devices would employ but still a much higher desalination capacity is obtained.

Whereas the NID cell uses cation-selective electrodes and an anion-exchange membrane, one can invert the concept by employing an anion-selective electrode and a cation-exchange membrane; therefore, we can call the latter in analogy to the NID technology “CID”, which stands for Cl-ion desalination. The first CID work predates even the NID work of Smith and Dmello^34^ in 2012, Grygolowicz-Pawlak *et al.* used a cation-selective membrane (sulfonated tetrafluoroethylene based fluoropolymer-copolymer; Nafion) coated around silver to achieve desalination of aqueous 600 mM NaCl.^30^ This work was adopted and modified for the field of microfluidics by Fighera *et al.* in 2017 when inkjet-printed Ag/AgCl was used as a counter electrode and working electrode for chip-scale water desalination (also using Nafion).^37^ While both these works show the desalination effect of the electrochemical operation of silver, their unique designs and reported experimental data do not allow to assess the desalination performance per electrode mass, cycling stability, and basic electrochemical features. The first CID work presented as an inverted NID system was explored in 2019. Arulrajan *et al.* used nanoporous carbon cloth without modification and after chemical surface-functionalization with (3-aminopropyl)triethoxysilane to enable an anion-selective response.^38^ This material pair was employed in a modified NID cell with a cation-selective membrane to achieve a desalination capacity of about 50 mg NaCl per 1 g of electrode material (53 mg g^−1^ = 0.91 mmol g^−1^) when operated in 20 mM NaCl at a cell voltage sweeping between −0.9 V and +0.9 V. Using the same operation parameters of Δ1.8 V per one CID cycle, the use of two non-modified carbon electrodes (*i.e.*, in the absence of enabled chloride sensitivity) yielded already a desalination capacity of 25 mg g^−1^. The latter value compares to reports on the same carbon in a conventional CDI cell operating at 0.9 V by division with a factor of 2; the resulting value of 12.5 mg g^−1^ is very close to 9 mg g^−1^ reported by Kim *et al.* at a lower molar strength of 5 mM NaCl.^39^ This work showed the versatility of the NID concept but was limited to low-molar strength and the use of carbon-based electrodes.

**Fig. 1:**
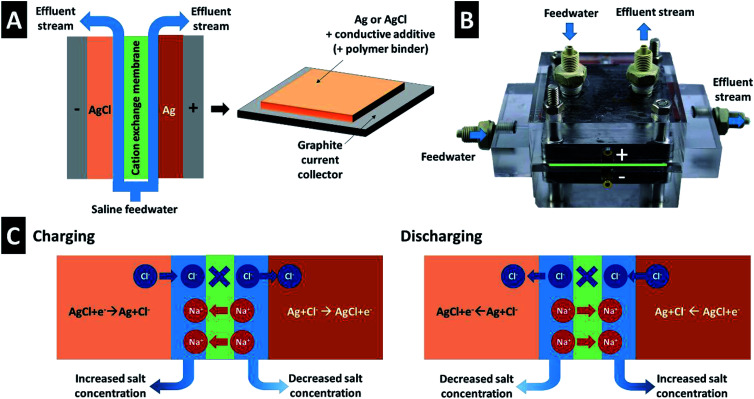
(A) Scheme of the CID cell and electrode configuration. (B) Photograph of the CID cell. (C) Concept of CID for electrochemical desalination.

In our work, we employed the CID cell based on Ag/AgCl conversion reaction and a polymer-based cation exchange membrane (Fig. 1). This illustrates how promising conversion reactions are for the desalination of seawater or other aqueous/saline media. We do so by using commercially available silver nanoparticles (AgNP) for one electrode, and AgCl obtained from electro-chlorination of AgNP as the other electrode. Therefore, the same Ag/AgCl conversion reaction, on forward- and backward direction, is the mechanism by which chlorine is removed. The overall desalination is then accomplished by charge-balancing of the aqueous media across a polymer-based cation-exchange membrane (Fig. 1C). Also, the use of AgCl in aqueous environments is motivated by the very low solubility of only about 13 μM.^40^ Such trace amounts of dissolved AgCl have a useful side effect per the antibacterial property of Ag^+^ ions to disinfect potable water.^41^ Here, we demonstrate the electrochemical response of each electrode separately and the use of the Ag/AgCl CID cell for operation in aqueous 600 mM NaCl. This concentration was chosen to illustrate the use of Ag/AgCl CID for seawater desalination but for an ideal saline medium which only contains NaCl. The cell delivers a very high desalination capacity of 115 mg g^−1^, even though a very small cell voltage of just Δ200 mV is used. This also translates to a low energy consumption of 2.5 kT per ion.

## 2. Experimental

### 2.1. Material synthesis and electrode preparation

Silver nanoparticles (AgNPs) (<200 nm, 99%, Sigma Aldrich) were used as a precursor for the silver chloride synthesis *via* electro-chlorination. Prior to AgCl synthesis, the AgNP electrodes were prepared by using two different carbon conducive additive: carbon black and multi-wall carbon nanotubes (CNT).

For AgNP electrode preparation, 80 mass% of the active material (AgNP) was blended with 10 mass% carbon black (C65, IMERYS) in isopropanol. Then, 10 mass% of polyvinylidene fluoride (Sigma Aldrich) was added and mixed with a pestle for 5–10 min in a mortar. *N*-Methyl-2-pyrrolidone (NMP) was dropwise added into the solid mixture while stirring until the slurry achieved a thick viscosity. The as-obtained slurry was doctor-blade casted on a graphite sheet (SGL carbon) with a wet thickness of 150 μm (dry thickness: *ca.* 100 μm). The coated electrodes were kept in the fume hood for one day and vacuum dried at 120 °C for 12 h. This electrode was used directly as it is and was labeled AgNP-10CB.

The AgCl electrode was derived *via* electro-chlorination from a binder-free buckypaper electrode composed of AgNP and multi-walled carbon nanotubes. For that, 200 mg of AgNP was mixed with 100 mL of ethanol and sonicated while stirring for 30 min. At the meantime, 50 mg of carbon nanotubes (CNT, Nanocyl NC7000) was mixed with 100 mL of ethanol and sonicated while stirring for 30 min to get a homogeneous suspension. After, the two suspensions were mixed and sonicated for 10 min. Aliquots of 50 mL of the mixed AgNP and CNT suspension were then vacuum filtered through the polymer membrane (Durapore filter, 220 nm pore size). After drying at 60 °C overnight, the free-standing AgNP-CNT electrode was obtained (100 μm thickness) by detaching it from the membrane. The resulting AgNP/CNT electrode had a total content of 20 mass% of CNT.

For the conversion of the AgNP/CNT into AgCl/CNT through an electro-chlorination process, disks with a diameter of 12 mm were cut from the electrode. The electrode was placed in a custom-built electrochemistry cell using spring-loaded titanium piston; for the setup, refer to ref. 42. To assemble the cell, the AgNP/CNT electrode was used as the working electrode. A 13 mm diameter of glass fiber mat (GF/A, Whatman) was employed as the separator. An oversized (50 mg) free-standing film of activated carbon (YP-80F, Kuraray; diameter: 12 mm) blended with polytetrafluoroethylene (PTFE) with 95 : 5 mass% (YP-80F/PTFE 95 : 5) was used as the counter electrode. 1 M NaCl was used as the electrolyte and chloride source at the same time. We connected the assembled cell with a VSP300 potentiostat/galvanostat (Bio-Logic) to apply chronoamperometry at 300 mV *vs.* Ag/AgCl for 3 h. The cell was disassembled, and the electrode was rinsed with deionized water for several times. The as-obtained chlorinated electrodes were labeled as AgCl-20CNT.

### 2.2. Structural characterization

Scanning electron microscopy (SEM) was carried out using a JEOL JSM-7500F system with an acceleration voltage of 3 kV. The samples were mounted on carbon sticky tape and analyzed without any conductive sputter coating.

X-ray diffraction (XRD) was conducted with D8 Discover diffractometer (Bruker AXS) with a copper source (Cu-K_α_, 40 kV, 40 mA), a Göbel mirror, and a 0.5 mm point focus. A two-dimensional VANTEC-500 detector covered an angular range of 20° 2*θ* with frames recorded at 20°, 40°, and 80° 2*θ* using a measurement time of 1000 s per frame. Rietveld analysis was carried out with the Bruker AXS software Topas 5.

### 2.3. Electrochemical characterization

Half-cell characterization was carried out with a custom-built cell using spring-loaded titanium pistons.^42^ To assemble the cell, a 12 mm disk of the electrode material (2–4 mg) was employed as the working electrode. A 13 mm diameter of glass fiber mat (GF/A, Whatman) was employed as the separator. A free-standing YP-80F/PTFE 95 : 5 electrode (thickness: 600 μm) was used as the counter electrode. The aqueous NaCl solution was inserted into the cell by vacuum filling with a syringe, and an Ag/AgCl reference electrode (3 M KCl, BASi) was located at the side of the cell body.

The cell was characterized by using VSP300 potentiostat/galvanostat (Bio-Logic). For cyclic voltammetry, the cell was cycled between −0.3 V and +0.3 V *vs.* Ag/AgCl at the scan rate of 1 mV s^−1^. For galvanostatic charge/discharge, the specific current of 0.1–10 A g^−1^ was applied to the electrode with the cut-off potentials of −0.3 V and +0.3 V *vs.* Ag/AgCl. The specific capacity of the electrode is calculated according to eqn (1).1
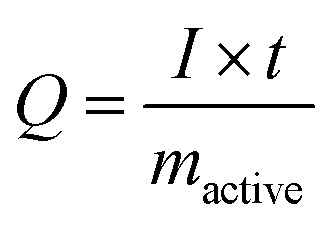
where *Q* is the specific capacity (mA h g^−1^), *I* is the current (mA), *t* is the time (h), and *m*_active_ (g) is the mass of either AgNP or AgCl (*i.e.*, the electrode mass without conductive additive and without polymer binder). To avoid any confusion between specific capacity (mA h g^−1^) and desalination capacity (mg g^−1^), we will use the term charge capacity for the specific capacity (mA h g^−1^) from here on forth.

### 2.4. Desalination performance

We used an inverted CID cell architecture for electrochemical desalination of aqueous 600 mM NaCl (Fig. 1A and B).^34,38^ Two flow channel compartments were separated by 180 μm thick cation exchange membrane (FKS10, FuMa-Tech). Our cell employed AgNP and AgCl as the positive and negative electrode, respectively. The cell was connected to the potentiostat/galvanostat VMP300 (Bio-Logic) using galvanostatic charge/discharge technique with the cut-off voltage of ±0.1 V and an applied current of 0.1 A g^−1^. Since the charge capacity of the electrodes does not remain constant, the duration of the desalination operation half-cycles varied from about 300 s (1st cycle) to about 200 s (25th cycle). A reservoir of 10 L of aqueous 600 mM was constantly flushed with N_2_ gas to remove dissolved O_2_. All measurements were carried out with a feedwater flow rate of 5 mL min^−1^.

The desalination capacity (DC; mg g^−1^) was calculated by use of eqn (2).2
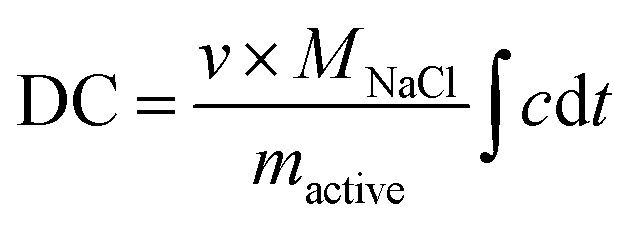
where *c* (in M) is the measured molar concentration, *t* is the time, *ν* is the volumetric flow rate (L s^−1^), *M*_NaCl_ is the molar mass of NaCl (58.440 g mol^−1^), and *m*_active_ (g) is the active electrode mass. The concentration of NaCl was recorded with a Metrohm PT1000 conductometric cell, and the pH values were recorded with a WTW SensoLyt 900P sensor probe.

The charge efficiency (CE; ref. 10) was calculated by eqn (3):3
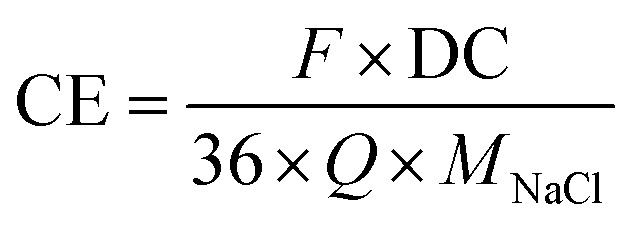
where *F* is the Faraday constant (96 485 A s mol^−1^), DC is the NaCl desalination capacity, 36 (=100/3600) is multiplied to *Q* for the unit conversion from mA h to Coulomb, *Q* is the measured charge, and *M*_Na_ is the molecular weight of sodium chloride.

## 3. Results and discussion

### 3.1. Structural characterization

**Fig. 2:**
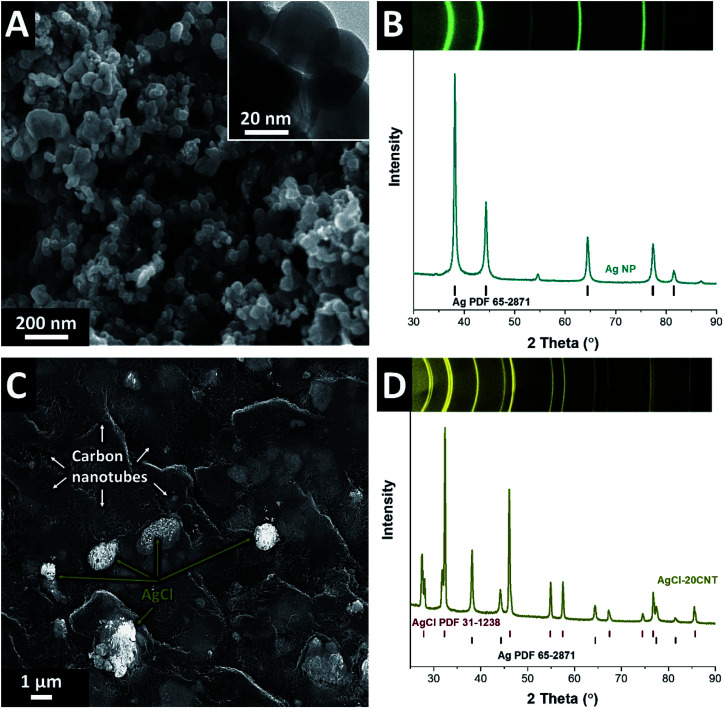
A) Scanning electron micrograph (inset: transmission electron micrograph) of the AgNP-10CB electrode and (B) X-ray diffraction pattern of the initial AgNP-10CB. (C) Scanning electron micrograph of the AgCl-20CNT electrode and (D) X-ray diffraction pattern of the initial AgCl-20CNT.

Fig. 2A shows scanning and transmission electron micrographs of AgNP blended with 10 mass% carbon black (AgNP-10CB). The well-dispersed AgNP agglomerates are composed of individual silver particles of around 50–60 nm. As confirmed by X-ray diffraction (Fig. 1B), the structure of AgNP is highly crystalline and face-centered cubic (space group *Fm*3̄*m*; ESI, Fig. S1†) with a lattice constant of *a* = 4.0897 Å and an average coherence length (domain size) of 75 nm (ESI, Table S1†). The finite domain size also visibly broadens the diffraction cones, as can be seen in the two-dimensional diffraction pattern (Fig. 2B).

As compared to the initial AgNP, the AgCl-20CNT shows a much coarser domain size with AgCl grains entwined within the CNT network (Fig. 2C). A domain size of more than 200 nm and the absence of peak broadening is confirmed by XRD (Fig. 2D). By use of Rietveld refinement, we have determined 85% conversion of Ag to AgCl in the AgCl-20CNT electrodes (ESI, Table S1†). Residual Ag in the AgCl-20CNT electrodes has also lost the nanoscale feature of AgNP and exhibits a lattice spacing of *a* = 4.0905 Å. AgCl is also cubic and has a unit cell parameter of *a* = 5.6200 Å.

### 3.2. Electrochemical characterization

**Fig. 3:**
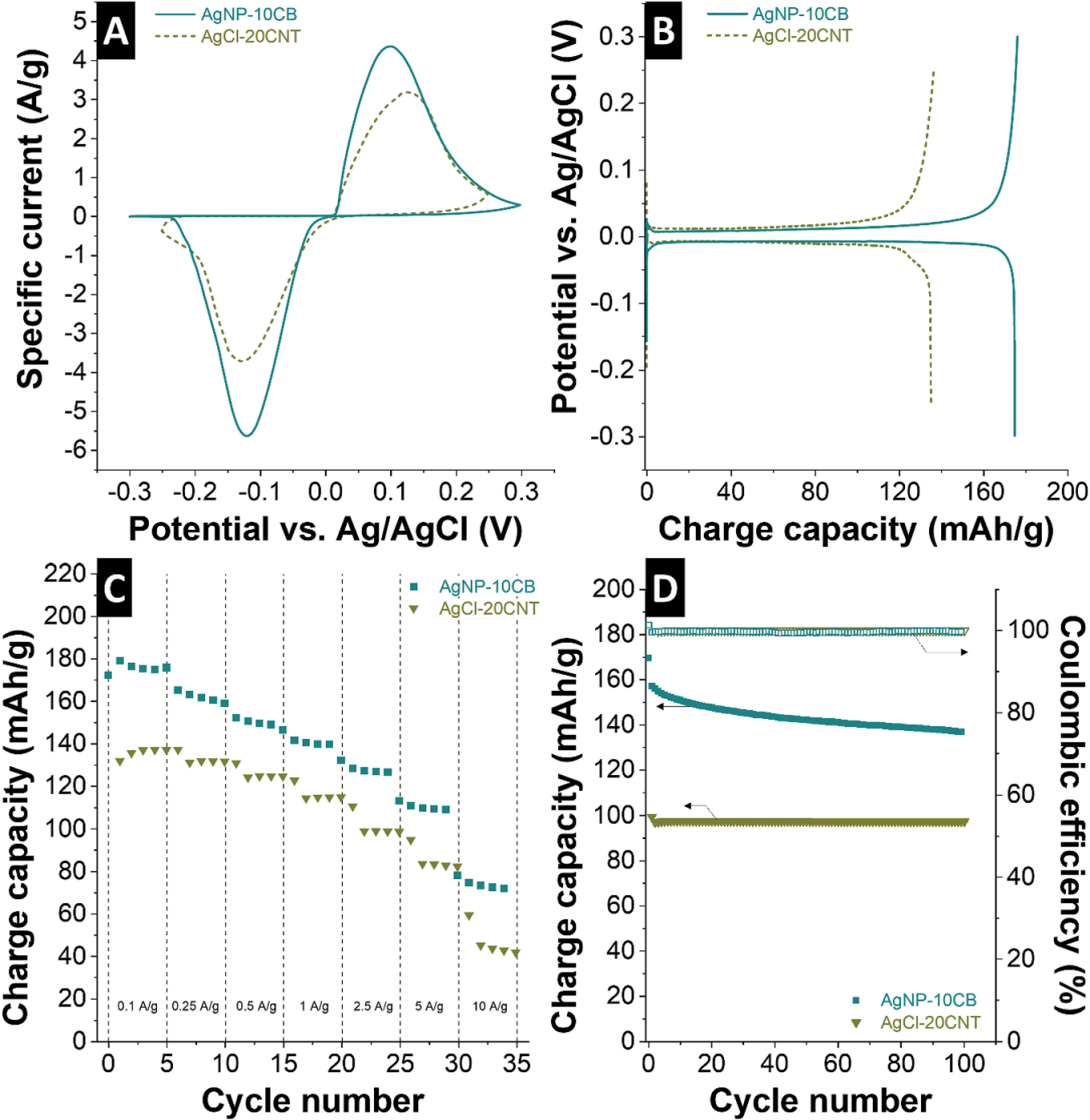
Electrochemical characterization using a half-cell setup and 1 M NaCl for AgNP-10CB and AgCl-20CNT electrodes. (A) Cyclic voltammetry at 1 mV s^−1^, (B) galvanostatic charge/discharge at 0.1 A g^−1^, (C) galvanostatic rate handling at 0.1–10 A g^−1^, and (D) galvanostatic charge/discharge cycling stability at 0.25 A g^−1^.

The characterization of the AgNP and AgCl electrodes in half-cell configuration was performed using aqueous 1 M NaCl to measure the basic electrochemical behavior and to quantify the charge storage capacity (Fig. 3). The overall reaction of Ag to AgCl and of AgCl back to Ag is well-known and given in eqn (4). Following this reaction, metallic silver converts to isolative silver chloride; therefore, our experiments ensured electrical conductivity by the presence of a conductive additive. In addition, the initially 15 mass% of Ag present in the electro-chlorinated AgCl-20CNT electrodes may also improve the charge transport process.

4 Ag_(solid)_ + Cl_(aqueous)_^−^ ⇌ AgCl_(solid)_ + e^−^

Cyclic voltammograms of AgNP-10CB and AgCl-20CNT half-cells are shown in Fig. 3A. As the AgNP-10CB electrode is scanned towards the positive potential and stopped at the cut-off potential of 0.3 V *vs.* Ag/AgCl, an oxidation peak at +101 mV *vs.* Ag/AgCl is observed, indicating the formation of AgCl. During the backward scan, the reduction process occurs at −121 mV *vs.* Ag/AgCl indicative of the reversible reaction of AgCl back to Ag. The AgCl-20CNT electrode shows broad peaks during anodic and cathodic sweeping, with oxidation and reduction peaks at +125 and −129 mV *vs.* Ag/AgCl, respectively.

The potential of oxidation and reduction potential characterizing the conversion reaction between AgCl and AgNP (eqn (4)) is explained by the Nernst equation (eqn (5) and (6)). Assuming a one electron transfer and high concentration, the formal potential (*E*_f_) can be shown as the standard potential (*E*_0_) that is between −10.5 mV and −10.6 mV *vs.* Ag/AgCl (eqn (3)).5
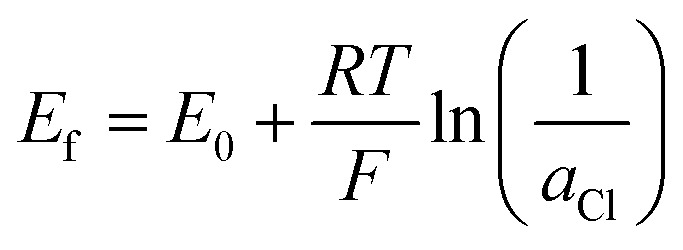
where is *E*_f_ is the formal potential, *E*_0_ is the standard potential (−10.5 mV *vs.* Ag/AgCl), *R* is the gas constant (8.314 J mol^−1^ K^−1^), *T* is the temperature (293 K), *F* is the Faraday constant (96 485 C mol^−1^), and *a*_Cl_ is the activity of chloride ion (equals 1 for high concentrations).6
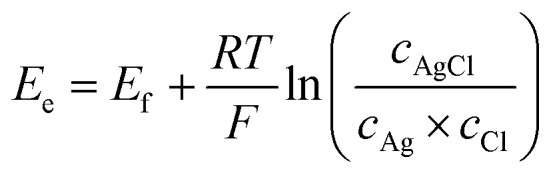
where *E*_e_ is the equilibrium potential, and *c* gives the concentration of either AgCl, Ag, or chloride ions.

The galvanostatic charge/discharge profiles (Fig. 3B) of AgNP-10CB and AgCl-20CNT in 1 M NaCl exhibit clear plateaus at the potential of ±50 mV *vs.* Ag/AgCl. This very narrow potential range should benefit desalination operation also within a narrow voltage window for the full-cell operation. The charge capacity of AgNP-10CB is 175 mA h g^−1^, which is about 70% of the maximum theoretical capacity of Ag being fully converted to AgCl. When cycling AgCl-20CNT, we obtained 140 mA h g^−1^ at 0.1 A g^−1^, which equals 77% of the theoretical maximum.

The overall rate handling of the AgNP-10CB and the AgCl-20CNT electrode is relatively high (Fig. 3C). This may be surprising for a conversion-type redox reaction. Of the initial capacity at 0.1 A g^−1^, AgNP-10CB maintains about 40% at the very high rate of 10 A g^−1^ (corresponding with a C-rate of about 40 C when considering the theoretical capacity of Ag). The AgCl-20CNT electrode also maintains about 30% of the 5th cycle capacity (and 32% of the 1st cycle capacity) at 10 A g^−1^ (corresponding with a C-rate of about 53 C when considering the theoretical capacity of AgCl).

As the last step for the basic electrochemical characterization, galvanostatic charge/discharge cycling was performed at a rate of 0.25 A g^−1^ (Fig. 3D). These measurements were carried out after cyclic voltammetry and galvanostatic charge/discharge rate handling; therefore, all electrodes had been cycled several times and, thereby, conditioned. Accordingly, AgCl-20CNT, which initially had shown a slight decrease of the charge capacity (Fig. 3C), provides a stable charge capacity of 100 mA h g^−1^ with no significant further decrease over the course of 100 cycles (Fig. 3D). The AgNP-10CB electrodes consistently provide a much higher capacity of 170 mA h g^−1^ in the first cycle; this capacity is gradually reduced to reach 137 mA h g^−1^ after 100 cycles (loss of 19%). Yet, the performance loss per cycles decreases over time. Post-mortem analysis of the AgNP-10CB electrodes after electrochemical operation in aqueous 1 M NaCl show that the performance decrease aligns with a potential-induced Ostwald ripening and coarsening of the particle size that leads to incomplete Ag/AgCl conversion; the corresponding X-ray diffraction data and electron micrographs are provided in ESI, Fig. S2.†

### 3.3. Desalination performance

Using the CID cell concept shown in Fig. 1, an AgNP-10CB and an AgCl-20CNT electrode pair was used for the desalination of aqueous 600 mM NaCl. The cell operation was carried out by cycling between −0.1 V and +0.1 V, to yield a net voltage difference of Δ200 mV. We chose this range based on the galvanostatic charge/discharge profiles seen in Fig. 3B. As can be seen, the plateaus for the Ag/AgCl conversion reactions are very flat, and material conversion occurs well below ±0.1 V *vs.* Ag/AgCl. The chosen narrow range demonstrates Ag/AgCl-based CID operation at a very small cell voltage. The AgNP-10CB and AgCl-20CNT electrodes were separated by two flow channels and a polymer-based cation exchange membrane. As AgNP is converted to AgCl, chlorine ions are removed from one flow channel, and charge compensation of the electrolyte is accomplished by transfer of one sodium ion across the ion exchange membrane into the other flow channel (Fig. 1C). The latter is enriched by sodium ions and, at the same time, by chlorine ions which are liberated as AgCl is being converted to Ag. Once both electrodes are fully converted to AgCl or Ag, the operation can be inverted; thereby, the flow channel with increased salt concentration becomes the effluent stream of desalinated water and *vice versa*.

**Fig. 4:**
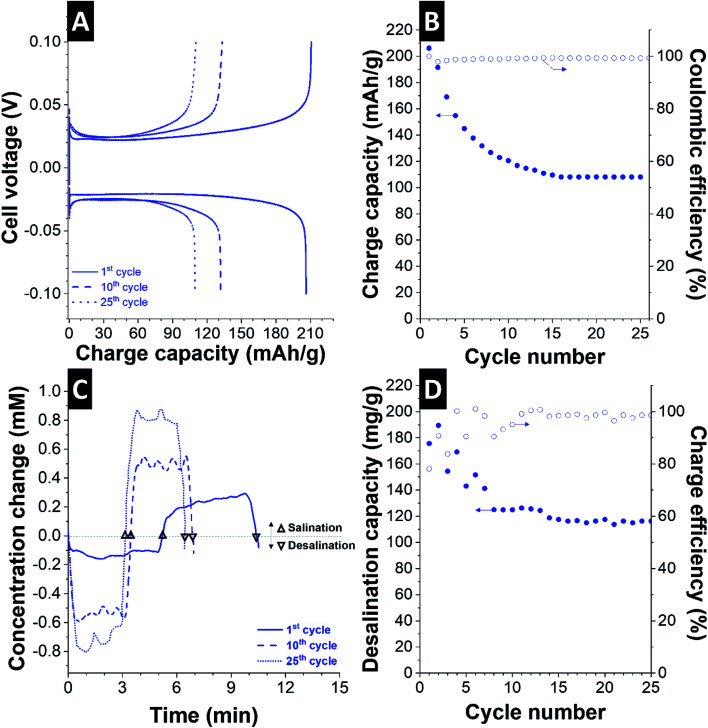
Electrochemical and desalination performance of AgNP-10CB/AgCl-20CNT CID cell in 600 mM NaCl using a cell voltage of Δ200 mV. (A) Charge/discharge profile of the 1st, 10th, and 25th cycle at 0.1 A g^−1^ and (B) corresponding cycling stability of the charge capacity. (C) Concentration change during operation and (D) corresponding desalination capacity and charge efficiency values.

We first measured the electric response of the cell. The characteristic galvanostatic charge/discharge profile for the 1^st^, 10^th^, and 25^th^ operation cycle of the CID cell is shown in Fig. 4A. The initial charge capacity of 205 mA h g^−1^ during charging is very close to the measured discharge capacity of 204 mA h g^−1^. There are also significant changes in the charge storage capacity of the CID cell over the course of 10 operation cycles (Fig. 4B), but the values stabilize thereafter at a level of 110 mA h g^−1^.

By inverting the cell polarity with a constant current of 0.1 A g^−1^, we see a drastic change in the concentration profile of the effluent stream (Fig. 4C). As desalination is accomplished, the concentration profile of NaCl first decreases before it goes back to the initial value as the electrodes assume their maximum state of charge. Polarity inversion converts AgCl back to Ag and Ag back to AgCl; accordingly, we see an increase in the concentration profile. The overall concentration change has an amplitude of about 200 μM NaCl for the first cycle and 600–700 μM NaCl at the 10^th^ and 25^th^ cycle. This desalination performance is consistent with the small electrode mass of 28 mg that yields the salt removal of 3.22 mg NaCl (*i.e.*, desalination capacity of 115 mg g^−1^). Hence, about 300 g of electrode material would be needed to lower the concentration of 600 mM to 10 mM NaCl for a water volume of 1 L in just one desalination step (and one *n*^th^ of this calculated mass when repeating the process *n*-times).

In alignment with the initial changes in the cell's charge storage performance, there are also changes in the desalination capacity (Fig. 4D). We obtained an initial desalination capacity of 176–190 mg g^−1^ in 600 mM NaCl. This very high performance correlates with the very high initial charge capacity in excess of 200 mA h g^−1^. Like the charge capacity, also the desalination capacity fades after a few cycles; thereby, we measured 125 mg g^−1^ after 8 cycles, and the performance stabilizes at about 115 mg g^−1^ after the 15^th^ cycle. This desalination capacity is accomplished at a desalination rate of 23 mg g^−1^ min^−1^ at the applied current of 0.1 A g^−1^. Concurrently, the initially fluctuating charge efficiency stabilized at a level of about 98% after the 15^th^ cycle. The desalination capacity fading is likely caused by the partially irreversible formation of AgCl at the positive electrode (AgNP-10CB electrode, Fig. S2†). This aligns with the decrease charge capacity half-cell performance during cycling of the AgNP-10CB electrode (Fig. 3D).

Based on the desalination performance, we can quantify the effective energy consumption per cycle. Cycling the AgNP-10CB/AgCl-20CNT CID cell in 600 mM between −0.1 V and +0.1 V removes a total of about 115 mg g^−1^ NaCl and consumes 97 μW h during charging. This value corresponds with an energy consumption of 2.5 kT per ion in 600 mM NaCl, which can further be reduced when considering recovered energy during discharging of the cell (92 μW h).

### 3.4. Charge-*vs.*-desalination correlation and comparison with other works

**Fig. 5:**
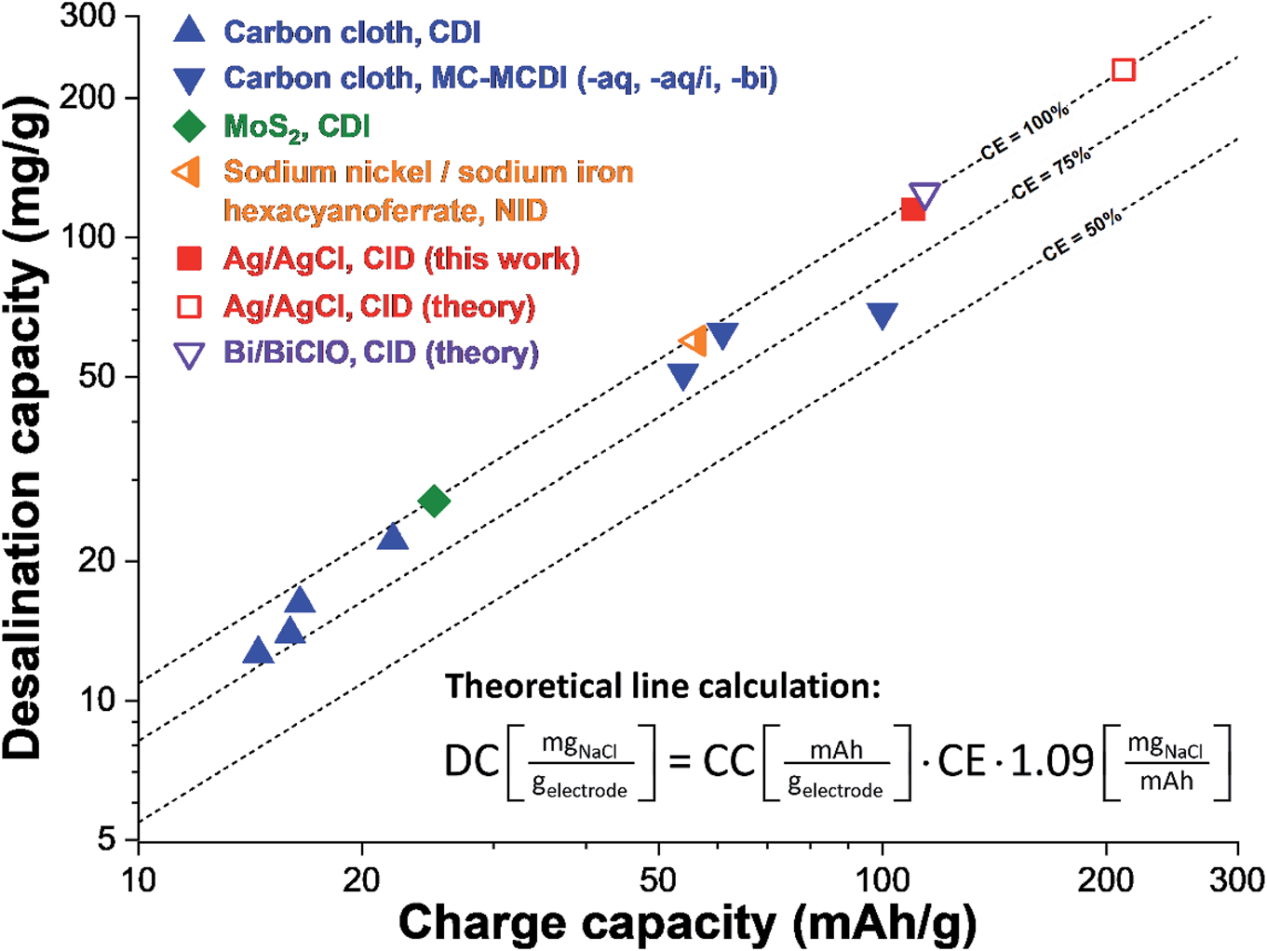
Charge-*vs.*-desalination (CVD) plot showing the universal correlation of charge capacity and desalination capacity. The graph shows data for different materials (carbons and non-carbons) and different cell geometries (including CDI, NID, and CID). The theoretical lines assume the stated values for the charge efficiency and are based on eqn (2) and (3). All values are tabulated and with references in ESI, Table S2†. Cell types: CDI (capacitive deionization), MC-MCDI (multi-channel membrane capacitive deionization), NID (sodium-ion desalination), CID (chlorine-ion desalination). Theoretical line calculation: CE (charge efficiency), CC (charge capacity), DC (desalination capacity).

As seen from eqn (2) and (3), there is a universal correlation between the invested electric charge and the number of removed salt ions. The charge-*vs.*-desalination (CVD) correlation is not losing validity when we compare different desalination cell concepts but is universally applicable (Fig. 5 and ESI, Table S2†). In contrast, the desalination capacity value alone may be misleading as the cell and material performance are strongly intertwined. Assuming a charge efficiency of 100% and ideal ion electrosorption with carbon electrodes, any increase of the cell voltage increases the charge capacity. This can be seen, for example, when we compare the desalination capacity of carbon cloth (Kynol 507-20) operated in a cell cycled between 0 V and 1.2 V (DC = 14 mg g^−1^) with the same material cycles between +1.2 V and −1.2 V in a CDI cell modified with ion exchange membranes (MCDI; DC = 22 mg g^−1^).^9^ The roughly doubled desalination capacity aligns with an increase in the charge capacity (adjusted for a single electrode) from 16 mA h g^−1^ to 22 mA h g^−1^ and an increase of the charge efficiency from 79% to 95%. Even higher charge capacity values are obtained for carbon when using multi-channel MCDI with concentration gradients or organic/aqueous bi-electrolytes system, with maximum values up to 54–61 mA h g^−1^.^9,43^

Redox-active electrolytes employing iodide may further enhance the charge storage capacity of carbon electrodes to about 100 mA h g^−1^ and the measured desalination capacity of 69 mg g^−1^ for a cell operated at 0.7 V aligns with a charge efficiency of 64%.^44^ This shows that the CVD plot is not limited to technologies based on ion electrosorption but can also be used to map the performance of non-carbon materials. For example, we see for MoS_2_ a desalination capacity of 27 mg g^−1^ in aqueous 600 mM NaCl which aligns with a charge capacity of 25 mA h g^−1^.^25^ The NID cell of Lee *et al.* employing nickel hexacyanoferrate and sodium iron hexacyanoferrate yielded a desalination capacity of 60 mg g^−1^ for 500 mM NaCl per the charge capacity of 56 mA h g^−1^.^36^

From the CVD plot (Fig. 5), we see that the Ag/AgCl desalination performance using cell voltage of just Δ0.2 V is much higher than using carbon electrodes, even when using cell voltages of Δ2.4 V or when using multi-channel concentration gradients or redox-active electrolytes. The performance of 115 mg g^−1^ at 110 mA h g^−1^ of our Ag/AgCl cell is near the theoretical value of the Bi/BiClO conversion reaction operated in a CID cell (125 mg g^−1^ at 114 mA h g^−1^). Yet, further optimization of the system has great potential for achieving even higher values. When considering the maximum possible charge storage capacity of a well-balanced Ag/AgCl system with 100% charge efficiency and a 100% degree of reversible conversion, we would expect a desalination capacity of 230 mg g^−1^ at a charge capacity of 211 mA h g^−1^. Such values, until now, have remained elusive for the fast-growing field of electrochemical desalination.

### 3.5. Remaining challenges for the Ag/AgCl CID concept

Our work has shown the very high desalination performance inherent to the Ag/AgCl conversion reaction process. Yet, grain coarsening and loss of charge capacity caused a corresponding drop of the desalination capacity. The latter stabilized at 115 mg g^−1^ which is superior to most materials explored for electrochemical desalination so far, especially when we consider the very small voltage window of Δ200 mV. However, 115 mg g^−1^ is only half of the theoretical value, and while it is unreasonable to assume 100% utilization of an electrochemical conversion process, there remains significant room for improvement. Clearly, the main electrode design goal must be on maximizing the amount of Ag and AgCl in the respective electrodes and to use as little conductive additive as possible. Homogeneous phase distribution is of high importance, and we provide in ESI† performance data of non-optimized AgNP and AgCl electrodes. Most specifically, future work will have to address the large loss of the charge capacity of the AgCl electrode and the significant particle coarsening.

## 4. Conclusions

Our work has demonstrated the facile use of silver nanoparticles paired with AgCl as a high-performance desalination battery with two flow-channels separated by a cation-exchange membrane. Such a chloride-ion desalination (CID) cell provides a desalination capacity far beyond the limit of electrosorptive carbon materials per the large charge capacity of the Ag/AgCl conversion reaction (theoretical maximum: 211 mA h g^−1^). When operated in high molarity aqueous solution of NaCl, electrodes based on AgNP yielded a charge capacity of 175 mA h g^−1^, while AgCl electrodes provided 140 mA h g^−1^ when operated at ±0.3 V at a specific current of 0.1 A g^−1^. These values translated in our Ag/AgCl-based desalination cell even at a very small voltage window of ±0.1 V (cell voltage of 200 mV) to a charge capacity of 110 mA h g^−1^ and a desalination capacity of 115 mg g^−1^ at a charge efficiency of about 98%. The low cell voltage of just Δ200 mV allows for very low energy consumption of about 2.5 kT per ion in 600 mM NaCl. Such high desalination capacity at such small voltages and low energy consumption have not been reported in the literature to the best of our knowledge. Yet, the Ag/AgCl system may even provide higher desalination performance with further optimization, especially regarding a higher degree of reversible AgCl electrode conversion.

## Conflicts of interest

There are no conflicts to declare.

## Supplementary Material

RA-009-C9RA02570G-s001
