# Supplementary material for: Low voltage operation of a silver/silver chloride battery with high desalination capacity in seawater
Source: RSC Adv. 2019 May 14;9(26):14849–58. doi: 10.1039/c9ra02570g (PMC9064245; doi:10.1039/c9ra02570g)
Supplement: RA-009-C9RA02570G-s001 [file RA-009-C9RA02570G-s001.pdf]

Electronic Supplementary Material (ESI) for RSC Advances.
This journal is © The Royal Society of Chemistry 2019

Supplementary Information

Low voltage operation of a silver/silver chloride battery with high desalination capacity in seawater

Pattarachai Srimuk,[1,2] Samantha Husmann,[1] Volker Presser[1,2,*]

[1] INM - Leibniz Institute for New Materials, 66123 Saarbrücken, Germany

[2] Department of Materials Science and Engineering, Saarland University, 66123 Saarbrücken, Germany

* Corresponding author's eMail: volker.presser@leibniz-inm.de

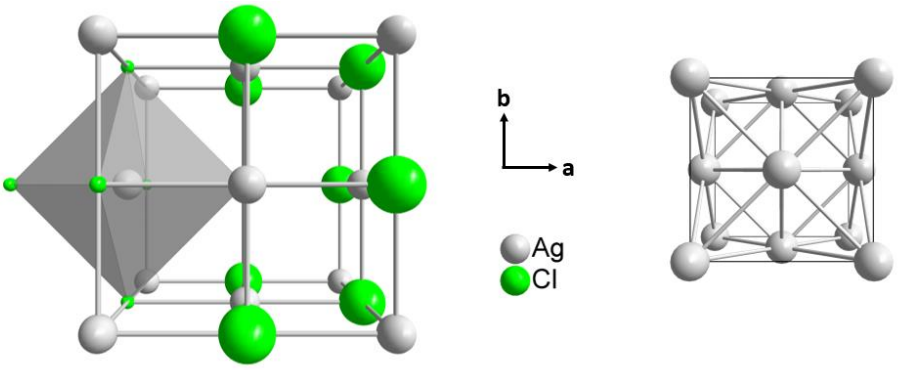

AgCl	Formula	Ag
225	Space group number	225
5.546 Å	Lattice constant	4.086 Å
3.922 Å	Shortest Ag-Ag distance	2.546 Å

Figure S1: Ideal crystal structure of AgCl and Ag along with selected crystallographic information.

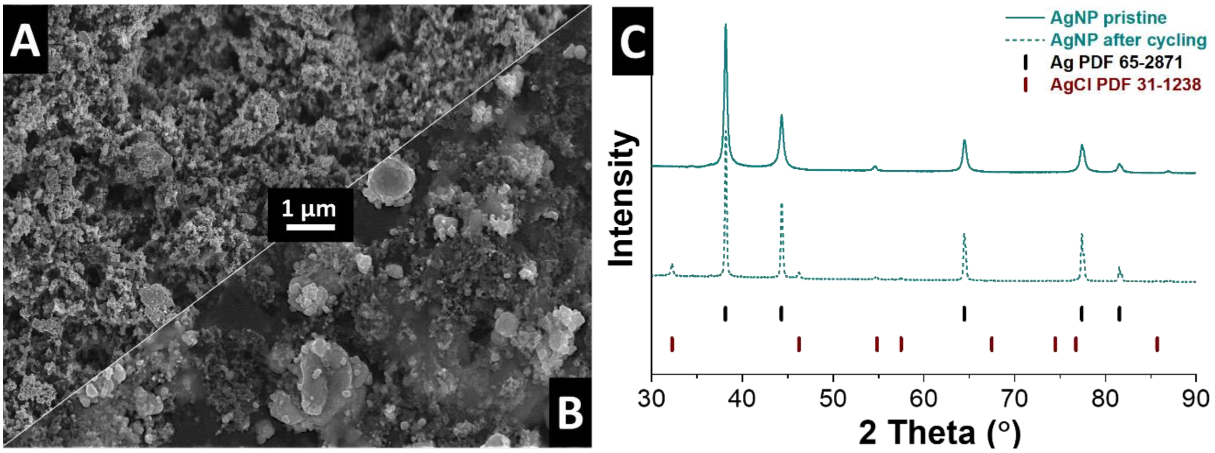

Figure S2: A) scanning electron micrograph of AgNP-10CB electrode before (up) and after electrochemistry (bottom) and B) X-ray diffraction pattern of AgNP-10CB electrode before and after electrochemistry.

Table S1: Measured crystal structure data obtained via Rietveld analysis of the measured X-ray diffraction pattern.

	Ag			AgCl		
	a (Å)	Size (nm)	Amount (%)	a (Å)	Size (nm)	Amount (%)
**AgNP**	4.0897	75	>99	-	-	-
**AgCl-20CNT**	4.0905	>200	15	5.6200	>200	85

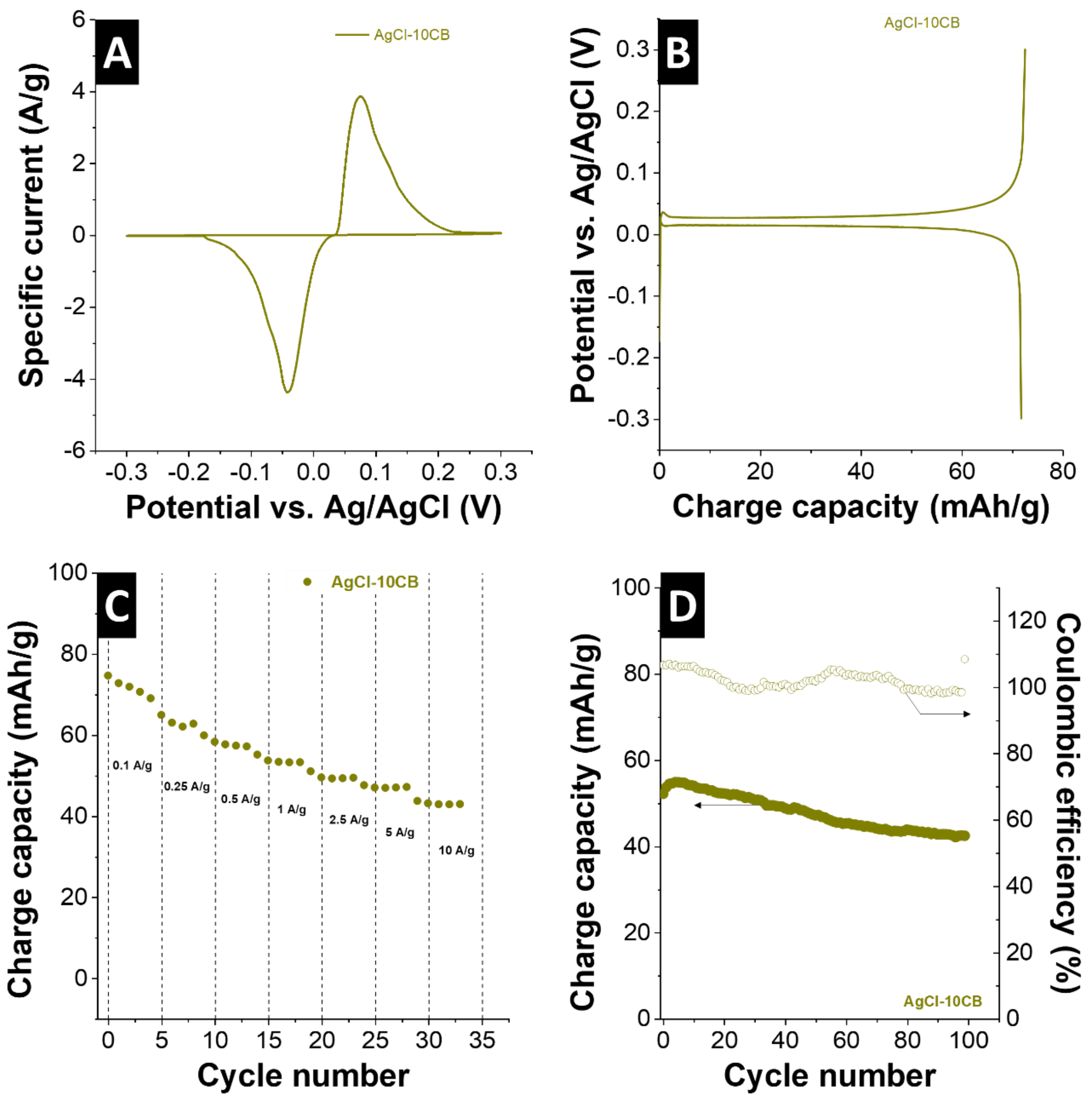

Figure S3: Electrochemical characterization using a half-cell setup and 1 M NaCl for AgNP-10CB. For this experiment, AgNP was blended with carbon black the same way as we did for AgCl-20CNT electrodes. A) Cyclic voltammetry at 1 mV/s, B) galvanostatic charge/discharge at 0.1 A/g, C) galvanostatic rate handling at 0.1-10 A/g, and D) galvanostatic charge/discharge cycling stability at 0.25 A/g.

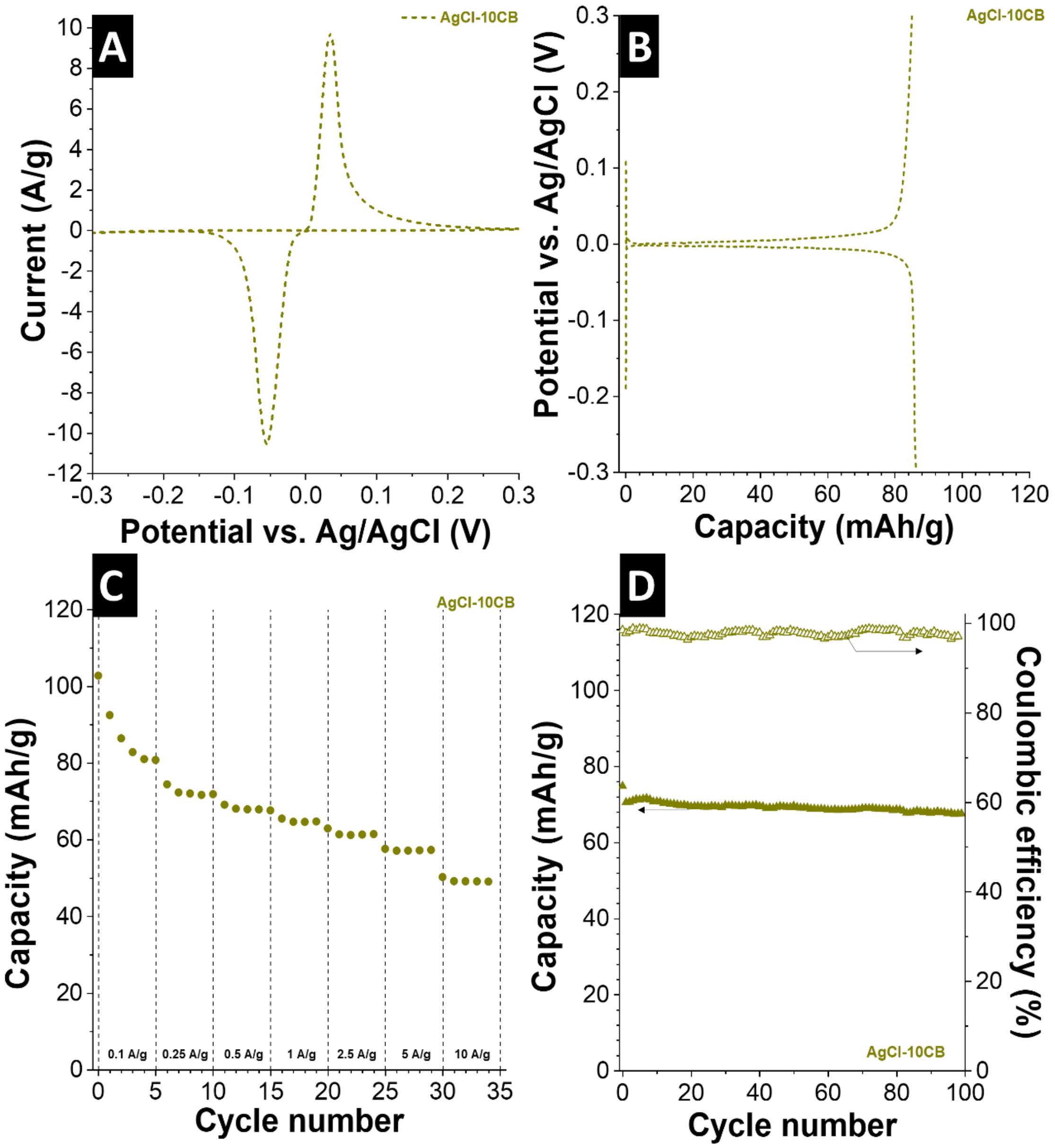

Figure S4: Electrochemical characterization using a half-cell setup and 1 M NaCl for AgCl-10CB. For this experiment, Ag was thermally chlorinated at 300 °C for 3 h, achieving full conversion to AgCl. The AgCl powder was then processes like AgNP-10CB electrodes to obtain AgCl-10CB electrodes. A) Cyclic voltammetry at 1 mV/s, B) galvanostatic charge/discharge at 0.1 A/g, C) galvanostatic rate handling at 0.1-10 A/g, and D) galvanostatic charge/discharge cycling stability at 0.25 A/g.

Table S2: Desalination performance and corresponding specific capacity for selected literature that provided required data on charge, charge efficiency, and salt removal capacity (considering NaCl as the only salt). NID: sodium-ion desalination. CID: chlorine-ion desalination; CDI: capacitive deionization; MCDI: membrane capacitive deionization. MC-MCDI: multi-channel membrane capacitive deionization (-aq: aqueous media; -bi: organic/aqueous bi-electrolyte).

Material	Cell type	NaCl concentration (mM)	Cell voltage (V)	Charge capacity (mAh/g)	Desalination capacity (mg/g)	Charge efficiency (%)	Comment
Any	Any	Any	Any	100	109	100	Theory, assuming Eq. 3
Any	Any	Any	Any	100	87	80	Theory, assuming Eq. 3
Any	Any	Any	Any	100	55	50	Theory, assuming Eq. 3
Ag/AgCl	CID	600	±0.1	110	115	96	This work
Ag/AgCl	CID	Any	Any	211	230	100	Theory, ideal
Bi/BiClO	CID	Any	Any	114	125	100	Theory, ideal
Kynol 5092-15	CDI	5	1.2	15[a]	13	76	Ref. [1]
Kynol 507-20	CDI	5	1.2	16[a]	14	79	Ref. [1]
Kynol 507-20+	CDI	5	1.2	17[a]	16	89	Ref. [1]
Kynol 507-20	MC-MCDI-aq[b]	5	±1.2	22[a]	22	93	Ref. [2]
Kynol 507-20	MC-MCDI-aq[c]	5	±1.2	54[a]	51	87	Ref. [2]
Kynol 507-20	MC-MCDI-bi[d]	5	2.4	61[a]	63	95	Ref. [3]
Kynol 507-20	MC-MCDI-aq/i[e]	100	0.7	100	69	64	Ref. [4]
MoS_2	CDI	500	0.8	25	27	96	Ref. [5]
Nickel hexacyanoferrate & sodium iron hexacyanoferrate	NID	500	Δ0.8[e]	56	60	98	Ref. [6]

[a] Charge storage capacity per one electrode for symmetric cells. [b] side-channel concentration: aqueous 5 mM NaCl. [c] side-channel concentration: aqueous 1000 mM NaCl. [d] side-channel concentration: aqueous 600 mM NaCl and 600 mM $NaClO_4$ in propylene carbonate. [d] side-channel concentration: aqueous 1000 mM NaI. [e] cell was cycled between 0.05 V and 0.85 V.

Supplementary References

1. C. Kim, P. Srimuk, J. Lee, S. Fleischmann, M. Aslan and V. Presser, *Carbon*, 2017, **122**, 329-335.
2. C. Kim, J. Lee, P. Srimuk, M. Aslan and V. Presser, *ChemSusChem*, 2017, **10**, 4914-4920.
3. C. Kim, P. Srimuk, J. Lee and V. Presser, *Desalination*, 2018, **443**, 56-61.
4. J. Lee, P. Srimuk, S. Carpier, J. Choi, R. L. Zornitta, C. Kim, M. Aslan and V. Presser, *ChemSusChem*, 2018, **11**, 3460-3472.
5. P. Srimuk, J. Lee, S. Fleischmann, S. Choudhury, N. Jäckel, M. Zeiger, C. Kim, M. Aslan and V. Presser, *Journal of Materials Chemistry A*, 2017, **5**, 15640-15649.
6. J. Lee, S. Kim and J. Yoon, *ACS Omega*, 2017, **2**, 1653-1659.
